# A qualitative study of barriers to and facilitators of optimal engagement in care among PLWH and substance use/misuse

**DOI:** 10.1186/s13104-016-2032-4

**Published:** 2016-04-22

**Authors:** Kamini E. Kuchinad, Heidi E. Hutton, Anne K. Monroe, Garrick Anderson, Richard D. Moore, Geetanjali Chander

**Affiliations:** Department of General Internal Medicine, Johns Hopkins Hospital, Baltimore, MD USA; University of Maryland School of Medicine, Baltimore, MD USA

**Keywords:** HIV, Substance use, ART, Adherence, HIV care continuum

## Abstract

**Background:**

Persons living with HIV (PLWH) and substance use/misuse experience significant barriers to engagement in HIV care at every step of the HIV care continuum including: (1) HIV testing and diagnosis (2) linkage to clinical care (3) retention in care pre-antiretroviral therapy (ART) (4) ART initiation and adherence (5) viral suppression. We qualitatively explored the facilitators of and barriers to participation in the HIV care continuum among PLWH with substance use/misuse.

**Methods:**

We performed semi-structured in-depth interviews with 34 PLWH in care with recent substance use. The transcripts were analyzed in an iterative process using an editing style analysis. Interviews were conducted until thematic saturation was achieved.

**Results:**

Participants attributed an escalation in drug use at the time of diagnosis to denial of their disease and the belief that their death was inevitable and cited this as a barrier to treatment entry. In contrast, participants reported that experiencing adverse physical effects of uncontrolled HIV infection motivated them to enroll in care. Reported barriers to retention and adherence to care included forgetting medications and appointments because of drug use, prioritizing drug use over HIV treatment and side effects associated with medications. Participants described that progression of illness, development of a medication taking ritual and a positive provider-patient relationship all facilitated engagement and reengagement in care.

**Conclusions:**

PLWH with substance use engaged in care describe barriers to and facilitators of optimal engagement related to and distinct from substance use. Greater understanding of the biologic, psychological and social factors that promote and impair engagement in care can inform interventions and reduce the increased morbidity and mortality experienced by PLWH with substance use.

## Background

Substance use among persons living with HIV (PLWH) can result in suboptimal engagement in the HIV care continuum such as: delayed diagnosis and/or entry into care, a lower probability of initiating antiretroviral therapy (ART), lower rates of retention in care, and viral suppression [1–6].

In the United States, individuals with substance use tend to be diagnosed with HIV and linked to care later in their disease course than nondrug users [2]. Similarly, both active and intermittent drug use is associated with a lower probability of initiating ART [3, 4]. Furthermore, HIV-infected drug users tend to have less cumulative time on ART and lower rates of retention in care over long periods of time [4, 7]. In a prospective study of 790 HIV-infected drug users, only 30.5 % of participants were continuously retained in care over a median of 8.7 years of follow up [4]. This lack of engagement in care is reflected in low rates of viral suppression [4, 8, 9]. In an analysis of 790 injection drug users (IDUs) in the the AIDS linked to the intravenous experience (ALIVE) study, a longitudinal study of IDUs in Baltimore, 53.9 % of IDUs achieved successful viral suppression, significantly lower than the nationally representative estimates of 72–77 % among the general population of individuals receiving HIV care [4]. In addition, a study of 1851 IDUs, found that active intermittent (OR 2.2, 95 % CI 1.4–2.9) and persistent drug users (OR 1.9 CI 1.2–2.8) had a significantly higher risk of opportunistic infection as compared to non drug users [8].

The impact of drug use on ART adherence has been seen across the spectrum of drugs including alcohol, cocaine, stimulants and heroin [10, 11]. However, substance use does not preclude successful engagement and retention in care. Indeed, studies demonstrate that HIV-infected individuals with drug use can have equal survival to those without drug use when prescribed and adherent to ART medications [12]. Therefore, understanding factors that can facilitate active participation in HIV care is essential in designing and implementing interventions to improve health care outcomes among this population.

The current literature demonstrates that substance use is a barrier to optimal engagement in the HIV care continuum. Few studies, however, have queried PLWH with substance use on their perceived barriers to and facilitators of their optimal engagement in HIV care [8, 10, 11, 13]. To date, studies examining the relationship between substance use and aspects of the HIV care continuum have been largely quantitative, describing poorer outcomes. Qualitative research can describe complex aspects of health care delivery that are not effectively described by quantitative methods [14, 15]. Qualitative approaches give voice to the individuals with substance use, and can provide deeper insight into factors that that affect optimal engagement in the HIV care continuum. Enhancing the literature on this topic through qualitative work can inform the development of future epidemiological work and practical interventions. Using semi-structured, in-depth interviews, the purpose of this study is to identify barriers and facilitators to engagement and retention in care among PLWH with substance use.

## Methods

### Study design, setting and participants

We performed semi-structured, in-depth interviews among individuals at least 18 years of age receiving care in an urban HIV clinic. These interviews were conducted between July 2013 and May 2014 as formative work for development of a behavioral intervention to reduce substance use among PLWH. We used purposive sampling recruiting individuals who were engaged in HIV care (at least one visit in the prior year), and who used opioids, cocaine, marijuana, or amphetamines in the 6 months prior to the interview. We deliberately chose individuals living with HIV with a recent past history of substance use. We reached out to providers to obtain referrals of individuals using specific drugs to ensure that we had an adequate range of drugs used. We also sought to recruit both light and heavy substance users. Specifically, we identified individuals in HIV treatment, as we believed that their stories could help inform substance use interventions delivered in HIV clinical settings.

### Procedure

Upon referral to the study, written informed consent was obtained. Participants then underwent the NIDA Modified Alcohol Smoking and Substance Involvement Screening Test (ASSIST). The ASSIST assesses both current and lifetime drug use of several substances including opioids, cocaine, marijuana, and amphetamine [16, 17]. Individuals reporting use of illicit substances over the past 6 months were eligible. No eligible participants who were referred refused to participate and no participant withdrew from the study. Participants were reimbursed $20.00 for their time and transportation costs.

Thirty-four, one-on-one interviews were conducted by either a female clinical psychologist (HH), a female physician (GC) and a male research assistant (GA) in private offices at the Johns Hopkins HIV Clinic. GC is a general internal medicine physician, with a specific focus on HIV and substance misuse. HH is a psychologist with a specialty in substance use, mood disorders and HIV. GA is a research assistant. Interviews were conducted in a private office in the clinic.

Interviews were conducted using semi-structured guides, which allowed for probing and clarification [18]. The guide, developed by investigators GC and HH, queried factors related to illicit drug use including: reasons for and factors influencing initiation and continuation of drug use; perceived pros and cons of use of specific drugs; drug use expectancies; triggers (internal and external); external influences on drug use; impact of HIV on drug use and conversely drug use on HIV care, including appointment and medication adherence; effect of drug use on doctor-patient communication; and strategies for reduction of substance use. The interviews were completed in about 60 min and were audio-recorded and transcribed verbatim. Identifying information was removed from interview transcripts. Participants were continuously enrolled until thematic saturation was achieved, at which point no new data were seen in the interviews. [19]. The institutional review board at the Johns Hopkins University School of Medicine approved the study.

### Data analysis

Interviews resulted in detailed narratives. For this project, we focused on how HIV infection may have affected individuals’ drug use trajectory, and how drug use may have affected individuals’ HIV trajectory. Qualitative analysis of the transcripts was done iteratively, in an editing style analysis. In an editing style analysis, conceptual units are identified in the text and used to develop themes [18, 20]. Data were organized in Microsoft Excel. Two investigators (GC and GA) independently read and coded the interviews. The first five interviews were read independently by each reviewer from the remaining interviews, and used to create a coding template. Each reviewer independently coded the remaining interviews using this template. One of the coders (GA) then took each independently coded document, compared the codes, and where there was disagreement, discussed with the other coder (GC) to obtain consensus. The research team identified and agreed upon any additions to the codebook that emerged after the development of the initial codebook. Overall agreement between the two coders was 90 %. The quality of our research methods was assessed against standards as defined in the literature [21].

## Results

Clinical and demographic characteristics of the 34 participants in this study are presented in Table 1. Nineteen (56 %) of the sample were men. The majority of individuals in this study had a history of polysubstance abuse (n = 25, 73.5 %) as reported on the NIDA ASSIST survey; the median NIDA ASSIST score of this sample was 28.5, (a score of 27, indicates high risk associated with substance involvement) [16, 17]. Themes are discussed within the framework of the HIV care continuum: linkage to care, retention in care and reengagement in care after periods of being lost to follow-up. Table 2 describes our major themes and the frequency with which these themes were reported.

**Table 1:** Demographic and clinical characteristics of interview participants

Characteristic	n (%)
Age, median	51
Sex	
Female	15 (44.1)
Male	19 (55.9)
Race	
African American	30 (88.2)
White	4 (22.8)
Drug use (lifetime)	
Polysubstance use	33 (97.1)
Single substance use	1 (2.9)
Drug use (past 3 months)	
No substance use	8 (23.5)
Single substance use	1 (2.9)
Poly substance use	25 (73.5)
Marijuana	21 (61.8)
Cocaine	17 (50.0)
Heroin	10 (29.4)
Methamphetamines	2 (5.9)
Injection drug use	
Never	14 (41.2)
Yes, but not in past 3 months	14 (41.2)
Yes	6 (17.6)
ASSIST substance involvement score, median	28.5
% Taking ART	88.3 %
Undetectable viral load (HIV-RNA < 400)	88.3 %

**Table 2:** Themes and subthemes that addressed barriers to and facilitators of engagement in care for HIV+ substance users

General themes and subthemes focusing on barriers and facilitators	n	Illustrative quotations
Linkage to care		
Barriers to linkage to care		
Denial of HIV diagnosis	8	“When I first found out, I was in denial like crazy. I wasn’t trying to hear it, you know, because I’m saying how can I, how could it be me….Yes, I used more often, it’s like that’s all I lived for was to get high”—female, age 47
Inevitability of death from HIV	11	“For me, I actually tried to run away again and I didn’t see [my doctor] for a while. I ran back into the streets because I found out, oh I had the bug of life, you know, my death is coming soon and everything. I wanted to escape, I ran away from home again…It did nothing for me, the shit was in me, it’s going to take me eventually”—male, age 33
Facilitators of linkage to care		
Illness/disease progression	8	“…they told me what my CD4 count was. And they said I was AIDS defined. And the doctor asked me did I want to die. I told him, no, I didn’t want to die… And that’s when I decided to get on the meds. From them telling me that I could die from it”—male, age 55
Retention in care		
Prioritization of drug use over HIV treatment	13	“When I drugged I ain’t gonna take my medicine right… I don’t want to do anything but get high. The way I was using, when I use, I would use”—female, age 51
Forgetting to take medication	10	“… when I’m high, I’ll forget. That’s put as simple as I can… I’m chasing the drug. I’m not thinking about my health.”—male, age 28
Side effects of medication	6	“…He would give me the medicine but I wouldn’t take it. And I was thinking like I knew a lot of people that had HIV too… when they wasn’t taking the medicine, they was fine, but as soon as they started taking the medicine, like they were dying like flies. So I figured I ain’t taking that medicine. I ain’t messing with that.”—female, age 42
Reengagement in care		
Illness/disease progression	8	“…, but I tried my best in spite of that to stay somewhat compliant with those pills, you know. I didn’t want to get sick, so I took them”—male, age 48
Ritual associated with medication	9	“At one point it used to make miss doses because I would be getting high. You know if I’m getting high, I wouldn’t think about taking my medication but now, you know, it’s like a program. Every time I eat a meal in the daytime and the evening time, I take my medication along with my meal so. As long as I eat two meals a day, I’m going to take my medication”—male, age 59
Positive provider-patient relationship	15	“… if there was something that she didn’t like that I’ve done, as far as taking my medication, she would argue with me and I wouldn’t have no problem with her doing that because she was right and I was wrong. That made me feel like she cared. So that’s how it was with me”—female, age 54

### Linkage to care

#### A diagnosis of HIV results in escalation of drug use and prevents linkage to care

Individuals perceived that their diagnosis of HIV led to increased drug use which prevented effective enrollment in care. Participants attributed this increase to 1. Denial at the time of diagnosis and 2. Thoughts that death from HIV was inevitable.

#### Denial and drug use to cope with an HIV diagnosis

Participants described avoidant coping, including denial and avoidance, and increased drug use after learning that they were HIV infected. Among those with experiences of denial, some participants expressed that drug use served as a reaction to the frustration and confusion they felt about contracting the disease. As one individual expressed: “When I first found out I was in denial like crazy. I wasn’t trying to hear it, you know, because I’m saying how can I, how could it be, me… I used more often, it’s like the ball rolling down the hill again. It was like that’s all I lived for was to get high” (Female, age 47).

For many participants, taking medication and engaging in care would be admitting that the diagnosis was a reality. By contrast, drug use allowed for the avoidance of these issues. One participant increased her drug use after her diagnosis due to denial of her diagnosis. She associated these feelings of denial to her lack of participation in care. “I went on a thing where I was like I really did want to think I had it, maybe it would go away …I didn’t even want to [go to the doctor] because I didn’t even want to believe I had it for one thing… You know, I just didn’t want to really believe it. Then… it took me a while to even start it taking my medicine right” (Female, age 51).

#### Escalation in drug use due to belief that death due to HIV is inevitable

Other individuals experienced a period of increased drug use post-diagnosis due to the belief that their death from HIV was inevitable. As one person explained, “For me, I actually tried to run away again and I didn’t see [my doctor] for a while. I ran back into the streets because I found out, oh I had the bug of life, you know, my death is coming soon and everything. I wanted to escape” (Male, age 33).

Some participants relapsed into drug use after periods of sobriety immediately after their diagnosis. One individual, who was abstinent from drugs at the time of diagnosis, stated: “from that point on, I think I wanted to use drugs again” (Female, age 61). Individuals related this fatalistic attitude about their prognosis to a lack of knowledge about the disease. One participant explained: “I was in denial when I found out. I started using even more… when I went back I started using again and at that point I never took medication for the HIV… I was really trying to destroy myself… I immediately thought it was a death sentence. You know, I didn’t get educated on anything. I started using even more” (Female, age 47).

Another participant agreed, stating that after his diagnosis:“… it was a real [messed] up day. And I started getting’ [messed] up after that day. … It increased 300 % [drug use] … Because I thought I was gonna be dead in a year and I said, what the [heck], get high. I was workin’ goin’ to school and everything, I had a career and a nice job, nice home, nice car, nice clothes, and everything. It all went up in smoke in a year, I didn’t care… I’m not gonna be here long. So get high, get high, get high, And that’s what I did for a year” (Male, age 53).

Beliefs about HIV infection as a rapidly terminal disease, and denial and avoidance of accepting this diagnosis led to escalation in drug use as a means of coping. This reaction to HIV diagnosis subsequently resulted in delayed linkage to care.

#### Adverse symptoms of HIV infection motivated enrollment in care

After diagnosis, individuals expressed that they were more likely to enroll in care once they experienced the adverse effects of an untreated HIV infection. When asked what motivated her to start taking medication after 10 years of being treatment naïve, one participant stated, “What changed my mind is how many times I started to get sick. …All during that 10 years, I was sick. I’ve had shingles. I’ve had TB. I’ve had pneumonia. I had a lot of stuff. So when I finally got here [to the clinic] … I was sick. I was close to dying and you know, and [the doctor] just worked with me. I finally decided to take the medicine and ever since then, I’ve been fine since and I take my medicine every day…I make all my doctor’s appointments” (Female, age 46).

Similarly, one man engaged in care after he was confronted with his prognosis without treatment. He states: “I was sick. I went to the emergency room and they ran a series of tests and they found out–they told me what my CD4 count was. And they said I was AIDS defined. And the doctor asked me did I want to die. I told him, no, I didn’t want to die. And that’s when the doctor explained to me that I needed to get on HIV meds or surely I was going to die. And that’s when I decided to get on the meds. From them telling me that I could die from it” (Male, age 55).

### Retention in care

#### Prioritization of drug use prevents ART and appointment adherence

Once participants were initially linked to care, they often faced difficulties successfully being retained in care, as defined by adherence to ART and appointments. Individuals, consumed by their drug use, neglected their medical care. Conscious prioritization of drug use over HIV treatment directly hindered both appointment and ART adherence. Indirectly, forgetting to take medication as a result of drug use prevented ART adherence.

Participants vividly described how their use impaired the ability to seek HIV care.

“… I didn’t make a lot of appointments. I wasn’t keeping doctor’s appointments. It’s like I just didn’t care… Because I was out all day and all night running and chasing and I wasn’t thinking about doctors. I wasn’t thinking about my health. I’ve been thinking about none of that, so yeah” (Female, age 42). For these participants, drug use superseded the need for medical care.

In addition to appointment adherence, individuals explained that ART was quickly forgotten once individuals started using drugs. One participant explained: “You going to forget because you running or chasing, or it’s just that you just don’t… F-the pills” (Female, age 41). Another participant succinctly summarized the effect drugs had on his engagement in care: “…when I’m high I’ll forget. That’s put as simple as I can…I’m chasing the drug. I’m not thinking about my health…So I wake up. I feel all right. I’m out the door. Then I realize I didn’t take my meds… [and then] I stay out ‘til I go back home and take them then” (Male, age 48).

This suboptimal adherence to ART was not only due to forgetting to take medication but also, a clear prioritization of drug use over HIV care. As one participant relates:“When I drugged I ain’t gonna take my medicine right… I don’t want to do nothing but get high. The way I was using, when I would use, I would use” (Female, age 51).

Other participants would not take their HIV medication because of the fear that the medication would interfere with the full effect of the high. One man explained “… when I knew that I was getting ready to relapse, I just stopped taking everything… I didn’t want the drugs to get in the way of me feeling the high” (Male, age 53).

Participants’ descriptions of their adherence while in the midst of active substance use, illuminate powerful and all consuming effect drugs can have in their lives.

#### Fear of adverse side effects of ART prevents adherence

Unrelated to substance use, participants described that they failed to adhere to ART because of the potential the adverse effects of these medications, both rumored and experienced. As one participant expressed, “[The doctor] would give me the medicine but I wouldn’t take it. And I was thinking like I knew a lot of people that had HIV too and […] when they wasn’t taking the medicine, they was fine, but as soon as they started taking the medicine, like they were dying like flies. So I figured I ain’t taking that medicine. I ain’t messing with that” (Female, age 42). Similarly, another individual had heard “…about people taking medication for it and they would be still sick, you know, so it was a lot of things that I didn’t know about…I heard things about the cocktails and how they would make them extremely sick, throwing up constantly, just where the regimen wasn’t working” (Female, age 47). Fearful of the rumored adverse effects of ART, individuals failed to either initiate or adhere to medication regimens.

In contrast, one woman avoided taking medications because of how they would change her physical appearance, and consequently other’s perception of her. She states that she “didn’t get on medication for years… because I was scared of how it would make me look. The people that I would see that were on AZT and different medications, they started looking like they were half-dead. You know, looked like they were ashy, black looking, hair started falling out, stuff like that … [I was worried about] how it would make me look and I didn’t want to tell anybody” (Female, age 50). The stigma of HIV infection and its treatment was enough to prevent adherence to medications.

Others stopped taking medications after experiencing significant side effects that severely impacted their quality of life. One participant explains “But I wasn’t takin’ no medicine. I would get the prescriptions, I might take it for a week, then I didn’t. They make me feel terrible, you know what I mean… Cause I’m not the same, I can’t do what I wanna do, I don’t work anymore, it takes me a week to clean up the bedroom and the bathroom, takes me two or three days to do it, but I get outta breath, I gotta sit down and so I said, f-it, I might as well get high” (Male, age 53).

When individuals reflected on factors that challenged their engagement in care, they identified factors applicable to the general HIV population at large, in addition to those related to drug use. While prioritization of drug use over HIV care negatively impacted adherence, fears and misconceptions about medication side effects and stigma surrounding the act of taking these medications also hindered adherence to care.

### Reengagement in care

#### Symptoms of untreated HIV infection motivated reengagement in care

Participants expressed that illness, or the feeling that one’s health was deteriorating, motivated them to reengage in both medication and appointment adherence after periods of being lost to follow up. The realization that their lives were actually in danger motivated patients to take control of their medical care. As one participant explains: “And they was like ‘Your cells are’ my cells was down to 27… yeah my CD4 count was down to 27. And she says ‘You know you’re full blown with AIDS now’ I said Oh hell, no. I need meds. I said I’m going to take it. I promise, I’m going to take it. So what they did, they made me come up here everyday” (Female, age 41). Another participant started adhering to medications “…because my count got real low. That’s when I started taking it and trying to do the right thing” (Female, age 51). Fear of illness often promoted medication adherence even during periods of active drug use. As one man explains, “Of course it wasn’t always six hours if I’m partying during everything too, but I tried my best in spite of that to stay somewhat compliant with those pills, you know. I didn’t want to get sick, so I took them” (Male, age 48).

#### Structure or ritual enabled effective medication adherence

Individuals perceived that establishing a ritual or structure around taking medications allowed for successful reengagement and retention in care. When patients were taking multiple medications, having a clear organizational strategy helped ease adherence to complex treatment regimens. For example, one participant depended on a pillbox to organize her complex medication plan. As she explained, “…it works for me…this is a ritual I do to make me on Sundays, do my medicine. Then I just pour them out on the week for two weeks, and when I know I’m running out, I redo them again” (Female, age 50). Taking medications at the same time everyday prevented patients from missing doses. As one individual described: “When I wake up I have habits and my habits, when I get up, the first thing I do is grab my pills and I plug up the coffee pot and I take my pills so. Before I even smoke a cigarette I’m taking my medicine, yeah” (Female, age 47).

Adopting medication into one’s daily routine promoted effective adherence even during periods of drug use. One participant explained how creating a ritual changed the way that drug use influenced his ability to take medication.

“At one point [drug use] used to make miss doses because I would be getting high. You know if I’m getting high, I wouldn’t think about taking my medication but now, you know, it’s like a program. Every time I eat a meal in the daytime and the evening time, I take my medication along with my meal so. As long as I eat two meals a day, I’m going to take my medication. If for some reason I miss a meal because I’m using that’s the very small percentage of the time that I would miss my medication but most of the time, even before I start using, I’ll make sure I take my–because I always take first–my morning dose” (Male, age 59).

#### Positive provider-patient relationships empowered individuals to participate in care

The provider-patient relationship plays a key role in maintaining medication and appointment adherence, especially after period of being lost to follow-up, thus contributing to successful reengagement in care. Individuals also expressed that satisfaction with their provider relationship positively impacted their desire to engage in care, to be truthful with their provider and to comply with treatment standards. Participants preferred providers that they described as “caring” and “open-minded”. Furthermore, participants were more likely to engage in care when they had providers who listened to their patient’s stories and explained the details of the disease and the treatment plan.

Many participants appreciated when their providers became upset with them when they did not follow treatment protocols because they viewed this reaction as a sign that their physician truly cared about their wellbeing. As one participant relays “… if there was something that she didn’t like that I’ve done, as far as taking my medication, she would argue with me and I wouldn’t have no problem with her doing that because she was right and I was wrong. That made me feel like she cared. So that’s how it was with me” (Female, age 54). The perception that their providers were invested in their health fostered a reciprocal relationship in which the patients became accountable for adhering to care.

In contrast, patients tended to disengage in care when they felt judged by their provider for their drug use or their HIV status. One individual believed that her substance use history negatively influenced the way her providers treated her. She explained: “A lotta times when you talk to a provider, and soon as they find out you’re an addict, everything, their whole demeanor changes. And I’ve had that done. Once you tell ‘em–if you tell ‘em you have a pain or ailment, the first thing they swear, you’re tryin to get drugs… they need to stop stereotypin’ their patients because of an addiction. It’s crazy, that makes people not wanna go to hospitals and don’t wanna see their doctors or anything else. And if somethin’ is wrong with them it makes them leery on sayin something about it because of the way they gonna be looked at” (Female, age 45).

Fear of illness, the establishment of a medication ritual and a positive doctor-patient relationship all motivated individuals to reinitiate and be retained in care during periods of both sobriety and substance use. These factors increased participants’ engagement in the care cascade resulting in improved HIV outcomes.

## Discussion

At an urban HIV clinic, we used qualitative interviewing to explore the barriers to and facilitators of engagement in care among 34 individuals with a history of substance use. From these interviews, themes related to the HIV care continuum emerged. The themes, reflecting linkage to care, retention in care and reengagement in care, characterized the participants’ relationship with the care continuum.

At the time of HIV diagnosis, participants described an escalation in drug use. Some individuals associated this behavior with feelings that death from HIV would be inevitable while others associated this increased drug use with feelings of denial about their disease. Theories of psychological adjustment suggest that an individual’s perspective on his or her disease process significantly impacts coping and adjustment to the disease [22]. Coping with chronic disease can involve approaching or avoiding demands of disease. In particular, avoidance is associated with cognitive and behavioral responses such as denial, suppression and disengagement [23]. Perceived threat to life goals and lack of disease-related self efficacy have been identified as key determinants of adjustment and have been associated with psychological pain and distress. For our participants, the diagnosis of HIV and its potential consequences resulted in significant psychological stress and maladaptive adjustment strategies. Drug use in particular may play a role in avoidance coping by diverting attention away from HIV diagnosis [22].

The experience of being diagnosed with a chronic illness such as HIV can either promote enrollment or engagement in care or can cause significant psychological stress and avoidant coping behaviors that can hinder linkage to care [24, 25]. The detrimental effect this experience has on enrollment in care can be exacerbated among already stigmatized populations. Among people who use drugs, increased psychological stress, due to any cause, can also lead to destructive behaviors and greater addiction vulnerability [26–28]. Our analysis suggests that this psychological stress manifests itself in increased drug use due to feelings of denial or inevitability of death associated with disease. Consequently, this phenomenon can prevent appropriate enrollment in care.

Case management interventions at the time of, and the period after diagnosis can help improve the likelihood that patients are linked to and retained in care [29, 30]. Current guidelines suggest that multiple case-management contacts for newly diagnosed individuals as well as intensive outreach for individuals not engaged in care within 6 months of diagnosis can help prevent individuals recently diagnosed with HIV from being lost to follow up [31, 32]. At the time of diagnosis, interventions should involve patient-centered communication in order to minimize any adverse outcomes associated with the experience of diagnosis. This type of intervention has been demonstrated to be effective in communicating the diagnoses of other diseases, such as cancer. In a cross-sectional study of Australian patients with melanoma, effective and conscientious communication of difficult diagnoses was associated with lower risk of anxiety and depression among patients [33]. As described in the cancer literature, patients’ preferred communication styles included focus on content (information about the disease), facilitation (setting and context of disclosure) and support [34, 35]. Intensive, repeated interventions at time of HIV diagnosis may be important to reduce morbidity and mortality among individuals with HIV and substance use, and decrease transmission risk through early engagement in care.

Participants identified barriers to optimal use of ART including (1) forgetting to take medications during periods of drug use, (2) prioritization of drug use over HIV medications and (3) adverse side effects of medications. Prioritization of substance use, over HIV medications has been described in the literature [36]. In a twelve-month prospective study of people who were on ART and used alcohol, participants missed doses or stopped medication altogether when using alcohol. Some individuals associated this behavior with beliefs that mixing ART and alcohol was dangerous. Others endorsed skipping medication doses because of beliefs that alcohol diminished the effectiveness of HIV medications, indicating a prioritization of substance use over treatment [37]. These beliefs are very similar to those expressed by our participants, reinforcing the importance of examining patients’ beliefs about interactions between ART and drugs when assessing their adherence to medications.

These barriers are not completely distinct from adherence barriers identified by nonsubstance using populations. A quantitative study of HIV-infected individuals in New York City identified forgetting appointments as one of the most common reasons for missing appointments. Our qualitative analysis corroborates this, and also suggests that missing appointments is associated with a conscious prioritization of drug use over HIV care [38]. Nonadherence to ART is attributed to a variety of structural, behavioral, and psychosocial factors amongst the nonsubstance using PLWH as well as the subpopulation of substance users.

Our analysis also identified facilitators to care that were similar to general HIV-infected population. Specifically, experiencing symptoms of untreated HIV infection motivated people to engage in care [39]. Furthermore, establishing a ritual or behavior pattern surrounding medication improved ART adherence [40]. In a meta-analysis of barriers and facilitators to ART adherence, patients reported that incorporating ART into daily routines and the use of reminder tools both helped improve medication adherence [39]. Evidence supporting educational counseling (brief medication adherence psychoeducational counseling sessions with multi-compartment weekly pill organizers) demonstrated a significant increase in medication and appointment adherence [38]. Though more research is needed on this topic, integrated programs that focus on integrated medication and behavioral therapy (including cognitive behavioral therapy, motivational interviewing) may be effective in improving treatment adherence [41]. Directly observed therapy has increased medication adherence and viral load suppression among substance users [42, 43]. In addition, studies suggest that social support plays a significant role in facilitating ART adherence [44].

The quality of the provider-patient relationship also impacts participation in HIV care. Emotionally supportive relationships have been associated with positive adjustment to chronic disease [22]. PLWH who perceived their primary care providers as empathetic, and knowledgeable about HIV expressed higher rates of satisfaction with their providers and higher rates of adherence to ART [45–47]. These individuals also reported that they felt comfortable speaking with their providers about personal issues [45]. A positive provider-patient relationship also significantly influenced engagement in care among HIV-infected substance users. A prospective study focused on medication adherence amongst injection drug users found that those who reported a positive provider-patient relationship had 45 % greater odds of ART use 6 months later [48]. Similarly, injection drug users who reported a positive relationship with their providers were significantly more likely to have an undetectable viral load. Of interest, participants associated episodes in which their providers became upset or frustrated at them over their lack of engagement in care as examples that their providers truly were invested in their care. This belief motivated individuals to be more accountable in their participation in the care continuum. Individuals were also motivated to adhere to care when their providers confronted them with potential consequences of their noncompliance with medical care. These findings contradict the non-confrontational approaches generally recommended in the literature, suggesting the need for further investigation [49, 50].

Conversely, negative provider-patient relationships can hinder engagement in the HIV care continuum, including initiation and adherence to ART. Illicit drug users treated by physicians with negative attitudes towards drug users tended to have later exposure to ART. Physicians may hesitate to prescribe ART to HIV-infected drug users because of the belief that these individuals will not adhere to treatment plans and that misuse of medication will lead to drug resistance [5]. This misperception can lead to increased morbidity and mortality amongst this population as well as increased risk of HIV transmission. Additionally, some studies suggest that provider emphasis on drug-related barriers to care and lack of emphasis on non-drug related barriers influences disparities in care amongst this population [38]. With appropriate structural and social supports, these individuals can achieve effective adherence [51, 52]. Directly administered antiretroviral therapy, medication assisted therapy, and multi-component nurse directed interventions all improved short-term adherence and virologic outcomes among HIV-infected individuals who use drugs [41].

This study has limitations. All of the individuals were recruited from one clinic. The majority of participants had a significant lifetime history of polysubstance use and reported substance use within the last 3 months prior to this study. Of note, these participants had varied levels of risk associated with substance use as defined by ASSIST scores. Nonetheless, the majority of participants in this study were adherent to ART and achieved viral suppression, reflecting successful engagement in the HIV care continuum. This characteristic of the study population may limit the transferability of our findings to those engaged in care; however, there are likely themes that transfer to other individuals with less severe substance use or use of a single substance; such as the utility of establishing a ritual for taking medications. Participants reflected upon their personal history of substance use and experiences with HIV to identify the barriers and facilitators that emerged as themes. As a result, we must consider recall bias as a potential limitation of this study. However, retrospective information gleaned from participants related to initial coping with receipt of a diagnosis, including denial, and escalation of drug use, may be applicable to those not engaged in care as yet, and provides insight into the types of support individuals may need at the time of diagnosis. Additionally, as is intrinsic to qualitative research, the interviewers may have indirectly affected the content of the interviews.

Our study provides deeper insight into the barriers and facilitators to care among PLWH who use drugs, specifically within the context of the HIV care continuum. Substance use can lead to increased HIV morbidity and mortality as well as increased risk of HIV transmission [53]. Therefore, lack of engagement in care for individuals who use drugs can have serious consequences for both individuals as well as the general population. While people who use drugs are often considered a distinct group within the HIV-infected population and thus have unique factors that influence their care, through our analysis, we also identified many barriers and facilitators to care that were similar to PLWH without a history of substance misuse. Our findings suggest the need for interventions that can improve participation in care at every step of the care continuum. Despite effective ART, engagement in the HIV care continuum remains suboptimal, resulting in increased morbidity, mortality, and disease transmission [54, 55]. Identifying these individuals and developing interventions to improve engagement in care is an essential part of reducing the burden of HIV infection.

## Conclusions

This study identified many barriers and facilitators to participation in care amongst HIV-infected individuals with substance use/misuse. After diagnosis, increased drug use hindered effective enrollment in care while illness motivated individuals to engage in care. Forgetting to take medications, prioritization of drug use over HIV care and adverse side effects of medications emerged as barriers to retention in care. Illness, ritual associated with medications and a positive provider-patient relationship served as facilitators to reengagement in care. These identified barriers and facilitators to engagement in HIV care can inform interventions to improve participation in care and thus reduce the morbidity and mortality associated with HIV infection amongst individuals who use drugs.
